# Inherited or Behavior? What Causal Beliefs about Obesity Are Associated with Weight Perceptions and Decisions to Lose Weight in a US Sample?

**DOI:** 10.1155/2014/632940

**Published:** 2014-09-23

**Authors:** Sasha A. Fleary, Reynolette Ettienne

**Affiliations:** ^1^Eliot-Pearson Department of Child Study and Human Development, Tufts University, 105 College Avenue, Room 104 Medford, MA 02155, USA; ^2^College of Tropical Agriculture and Human Resources, University of Hawaii at Manoa, 1955 East West Road, AgSci 216, Honolulu, HI 96822, USA

## Abstract

*Objectives.* To identify the extent to which (1) beliefs about obesity and obesity-related behaviors distinguish individuals based on weight perception (WP) and (2) beliefs about obesity predict perceived health status and WP and how these in turn predict decisions to try to lose weight. *Method.* 7456 noninstitutionalized US adults (M_age_ = 54.13, SD_age_ = 16.93; 61.2% female; 75.9% White) completed the 2007 Health Information National Trends Survey. Multinomial logistic regressions and structural equation modeling were used to accomplish study objectives. *Results.* Age, gender, information-seeking, health status, belief that obesity is inherited, and knowledge of fruits and vegetables recommendations distinguished participants based on WP. Beliefs about obesity predicted health status, WP, and trying to lose weight in the general model. The models varied based on gender, race/ethnicity, education, and weight misperception. *Conclusion.* This study supports the role of beliefs about obesity, WP, and health perceptions in individuals' decisions and actions regarding weight management. This study increases our understanding of gender, race/ethnicity, education, and weight misperceptions differences in decisions to lose weight. This knowledge may lead to targeted interventions, rather than “one size fits all” interventions, to promote health and prevent obesity.

## 1. Inherited or Behavior? What Causal Beliefs about Obesity Are Associated with Weight Perceptions and Decisions to Lose Weight in a US Sample?

Obesity is a growing public health concern in the United States (US) and worldwide [1] with rates doubling among adults between 1980 and 2004 [2, 3]. Though no significant increase in prevalence rates has been noted since 2004, about two-thirds of US adults are overweight or obese [4]. Biological contributions to obesity include thyroid disease [5] and genetic predisposition [6], while major modifiable behavioral contributions include diet, physical activity (PA), and sedentary activity. Research has shown that self-perceptions and beliefs about weight status [7, 8] as well as beliefs about the origins of obesity, influence individuals' behaviors to improve their weight status [9].

In a study comparing weight perception (WP) to body mass index (BMI), Dorsey et al. [10] found that 40% of underweight and overweight participants and 8% of obese participants misidentified themselves as “about the right weight.” Bennett and Wolin [11] and Dorsey and colleagues [10] found significant weight misperceptions among ethnic minorities and individuals with lower education levels. Several researchers have found that WP is just as important, if not more important, than actual BMI in influencing obesity-related behaviors and perceive it as a barrier to reducing obesity [12, 13]. Additionally, Anderson et al. [14] found that, among overweight and obese women, those who were dissatisfied with their body were nine times more likely to try to lose weight. This is particularly important since misidentification of weight status may lead to higher or misinformed body satisfaction, lower perceived need for weight management behaviors, and lower motivation to try to lose weight. This study builds on other studies by exploring correlates of WP beyond demographic variables. This study also attempts to further understand how WP is related to trying to lose weight.

Besides WP, researchers also debate the extent to which information on the genetic contributions to obesity is helpful in addressing the obesity epidemic [15]. Recent research and Genome Wide Association Studies have found more than 120 potential obesity-related genes [16] with 22 replicated in multiple populations [17]. Obesity research has focused primarily on the KAB model (changing knowledge, which leads to changes in attitudes and this leads to changes in behavior) to explain weight loss decisions. However, in this context, it is uncertain whether knowledge of genetic markers for obesity is correlated with increased or decreased preventive health and weight management behaviors. As we continue to explore this, we believe that it is imperative to get a baseline understanding of the relationship between WP and beliefs about the origins of obesity as well as obesity-related behaviors (notably, knowledge of fruit and vegetable [F&V] and PA recommendations). Understanding this will provide insight on the differences in thought processes of people in different perceived weight classes and ultimately inform interventions to reduce obesity.

The purpose of this paper is twofold. First, we aim to identify the extent to which beliefs about the origins of obesity and obesity-related behaviors distinguish people based on WP. Second, we examine the extent to which beliefs about the origins of obesity predict perceived health status and WP and how these in turn predict individuals' decisions to lose weight.

**Figure 1:**
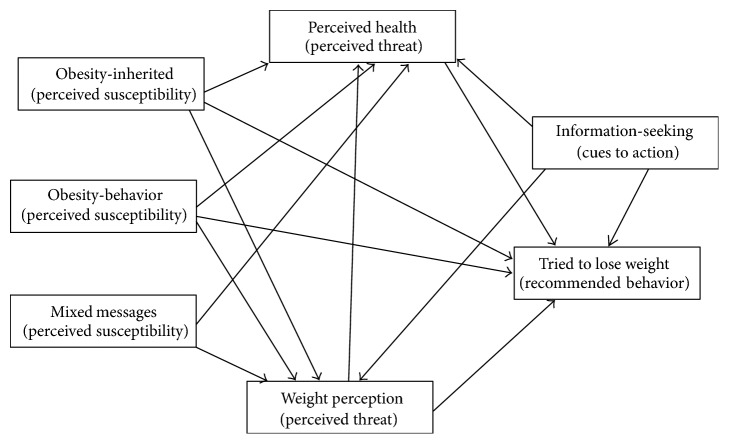
Hypothesized Health Belief Model paths measured with structural equation modelling.

The tenets of the Health Belief Model (HBM) were utilized to explore the extent to which causal beliefs about obesity are related to decisions to lose weight. The HBM is similar to decision-making theories since it focuses on a goal, personal motivation, and probability of attaining that goal [18]. The HBM assumes that individuals will not take preventive or seek treatment measures unless they have some level of knowledge and motivation, see themselves as being vulnerable, and perceive the condition as threatening. Furthermore, individuals must believe an intervention to be beneficial and identify few difficulties in implementing the intervention [19–21]. For this study, tenets of HBM were used to explore the extent to which perceived susceptibility (causal beliefs about obesity) was related to perceived threat (weight perception and health status) and whether this was related to trying to lose weight (Figure 1). Cues to action were measured by participants seeking information on the internet about diet and PA, while demographic variables were treated as modifying factors. Note that Figure 1 does not show correlations reported in Table 3 or paths controlling for gender and race/ethnicity reported in Table 4.

## 2. Method

### 2.1. Procedure

Data for this study were taken from the 2007 Health Information National Trends Survey (HINTS), a national survey conducted by the National Cancer Institute (NCI) among noninstitutionalized US adults [22]. Recruitment for the 2007 HINTS data was done via random digit dialing (RDD) and mailings sent to random US mailing addresses. The 2007 HINTS sample completed either a one-time telephone or paper and pencil survey. Overall response rates for the mail mode were 31% and 24.2% for the RDD mode [22]. More information regarding HINTS and the general methods utilized can be found on the HINTS website (http://hints.cancer.gov/).

### 2.2. Participants

The total 2007 HINTS sample consisted of 7674 adults; 3582 completed the survey by mail and 4092 completed the survey by telephone. Two hundred and eighteen participants were excluded due to missing data for the obesity beliefs, WP, and BMI variables and this resulted in a final sample size of 7456.

### 2.3. Measures

#### 2.3.1. Demographics

Participants provided their age (M = 54.13, SD = 16.93), race/ethnicity, gender (male = 1, female = 2), and education. BMI (kg/m^2^) was calculated and provided by HINTS and was based on participants' self-reported weight and height. For the purposes of this study, participants were assigned to the following categories:* underweight* (BMI = less than 18.5; *n* = 130),* normal weight* (BMI = 18.5 to less than 25; *n* = 2550), and* overweight* (BMI = 25 or greater; *n* = 4562). Obese individuals (BMI > 30) were included and analyzed in the overweight category.

#### 2.3.2. Obesity Beliefs

Participants were asked one question about mixed messages about the effect of weight on health: “There are so many different messages about whether being overweight is harmful to one's health it is hard to know what weight one should maintain to be healthy. Would you say you…?” (labeled mixed messages). Participants responded on a 4-point Likert scale from* strongly disagree* to* strongly agree* (M = 2.84, SD = 1.04). Participants also answered two questions about their beliefs on the cause of obesity including behavioral (M = 3.70, SD = 0.57) and inherited (M = 2.80, SD = 0.86): “To what extent do you believe that obesity is caused by overeating and not exercising?” (labeled obesity-behavior) and “To what extent do you believe that obesity is inherited?” (labeled obesity-inherited). For both items, participants responded on a 4-point evaluation scale ranging from* not at all* to* a lot*. The correlation between these two items was not significant (*r* = −0.02, *p* = 0.10), hence the need to examine each question separately.

#### 2.3.3. Weight Perception

Participants' WP was elicited via the following question: “Right now do you feel you are…” and answer choices included* underweight*,* slightly underweight*,* just about the right weight for you*,* slightly overweight*, and* overweight*. Participants who perceived themselves as underweight (*n* = 109) and slightly underweight (*n* = 227) were combined into one category, underweight, due to small sample sizes.

#### 2.3.4. Weight Misperception

Several steps were taken in order to calculate weight misperception. BMI was analyzed in categories using the criteria applied above (see Section 2.3.1) with* underweight (BMI < 18.5)*,* normal weight (BMI 18.5*–*<25)*, and* overweight* (all participants with BMI > 25). Secondly, WP (see Weight Perception section) was also collapsed into three categories for ease of analysis and to match BMI categories by (1) combining* slightly underweight* and* underweight* into* underweight*, (2) combining* slightly overweight* and* overweight* into* overweight*, and (3) leaving* just right weight* as is but relabeling it as* normal weight*. Next, WP was subtracted from BMI and the negative values were combined and coded as* overestimate* (*n* = 875, 85% female, 80% White), the positive values were combined and coded as* underestimate* (*n* = 849, 36% female, 64% White), and 0 was coded as* correct estimate* (*n* = 5493, 60% female, 77% White).

#### 2.3.5. Fruit and Vegetable (F and V) Recommendations

Participants identified how many servings of F and V they thought a person should eat daily for good health via an open ended question. Responses ranged from 0 to 50 and responses 20 and above (*n* = 7) were considered outliers and excluded from further analyses. Participants' responses were recoded into less than 5, 5–9, and more than 9.

#### 2.3.6. Physical Activity (PA) Recommendations

Participants were asked to identify the recommended number of days per week PA is required (range 0–7 days) as well as how long (time in minutes per day) adults should engage in daily PA (range 0–570 minutes). Responses for number of days were multiplied by number of minutes per day. Participants' responses were recoded into five categories: (a) inactive or 0 activity; (b) less than recommended or 15–149 minutes per week; (c) minimum recommended or 150 minutes per week; (d) more than recommended or 151–300 minutes per week; (e) more than 300 minutes per week. These categories/coding are based on the current recommendations of the American College of Sports Medicine to achieve 150 minutes of moderately intense or 75 minutes of vigorous PA a week [23].

#### 2.3.7. Health Status

Participants indicated their perceived health status (PH) on a 5-point evaluation scale ranging from* poor* to* excellent* (M = 3.41, SD = 0.96).

#### 2.3.8. Information-Seeking Behavior

For information-seeking behavior, participants indicated if they used a website to help with their diet, weight, or PA in the past 12 months. This variable was coded (2) for yes and (1) for no.

#### 2.3.9. Weight Loss Behavior

Participants were asked if they tried to lose any weight in the past 12 months. This variable was dichotomized (0 = no, 1 = yes).

### 2.4. Statistical Analyses

Descriptive statistics were calculated. The extent to which differences in causal beliefs about obesity, recommended F and V and PA behavior, and demographic variables distinguished participants of differing WP was obtained using multinomial logistic regressions. Structural equation models (SEM), that included group specifications for gender, race, education, and misidentified weight status, were used to test the HBM paths in Figure 1. The path between mixed messages and trying to lose weight was originally considered in the model but was set to 0 due to nonsignificance in all the models tested. Additionally, gender and race/ethnicity were controlled for in the misidentified weight status model. Descriptive statistics and regression analyses were computed using SPSS 16.0 [24]. SEM was conducted using AMOS 16.0 [24].

## 3. Results

### 3.1. Sample Characteristics

Descriptive statistics for demographics and categorical variables are presented in Table 1. Participants varied significantly on age, race/ethnicity, education, gender, and WP.

### 3.2. Correlates of Weight Perception

The results of the multinomial logistic regression analysis (*Nagelkerke R* ^2^ = 0.21; *χ* ^2^ = 897.61, *df* = 60, *P* < .001) examining the extent to which beliefs about obesity, demographic variables, and recommended health behaviors distinguished participants based on WP are presented in Table 2. The usefulness of this model was measured by the proportional by chance accuracy rate (125% improvement = 40.01%). The regression successfully classified 54.3% of participants; therefore the model was useful. Age, gender, race/ethnicity, education, obesity-inherited, mixed messages, PH, information-seeking, and recommended F and V were significant in the likelihood ratio tests. Obesity-behavior and recommended PA were not significant in the likelihood ratio tests; therefore these ratios were not interpreted further.

Participants who perceived themselves as underweight, “just right,” and slightly overweight tended to be younger, male, less likely to engage in information-seeking, and more likely to view their PH as better than participants who perceived they were overweight. These participants were also more likely to be Asian than White when compared to overweight participants. Furthermore, these participants were less likely to endorse obesity-inherited than overweight participants. They were also more likely to underestimate the recommended levels of F and V consumption than overweight participants. Note that participants who perceived themselves as underweight were more likely to underestimate or overestimate the recommended daily F and V when compared to overweight participants. Participants who perceived their weight as “just right” or slightly overweight were more likely to be college graduates rather than have some college education than participants who perceived themselves as overweight. Additionally, compared to overweight participants, participants who viewed themselves as slightly overweight were more likely to be college graduates than high school graduates.

### 3.3. Tenets of HBM Predicting Decision to Lose Weight

**Figure 2:**
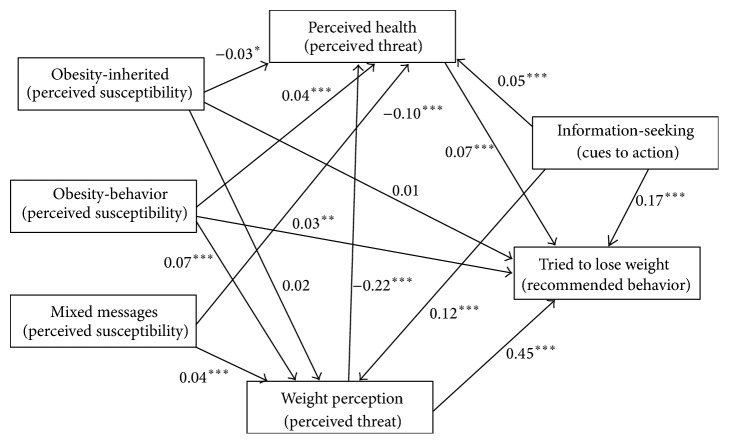
Significant hypothesized Health Belief Model paths for the general model.

The SEM results predicting participants' decision to lose weight are presented in Table 3. The general model (Figure 2), which does not include demographics, was a good fit to the data (*χ* ^2^ = .30, *P* = .59; CFI = 1.00; RMSEA = .000). For the general model, mixed messages, obesity-inherited, and information-seeking were positively predictive of WP. Additionally, information-seeking, WP, and all causal beliefs about obesity predicted PH with negative relationships noted for mixed messages, obesity-inherited, and WP. Regarding trying to lose weight, obesity-behavior, information-seeking, WP, and PH were predictive of the behavior. Although the general model had good fit, we tested group models for gender, race, and education since these are modifying factors according to the HBM. Any deviations from the general model are explained in the following subsections.

#### 3.3.1. Gender

This model had good fit (*χ* ^2^ = 2.81, *P* = .06; CFI = .99; RMSEA = .016). Obesity-behavior did not predict WP in the general or female models but was significant in the male model. Obesity-inherited and obesity-behavior predicted PH in the general and female models but were insignificant in the male model. Obesity-behavior predicted losing weight in the male and general models but was insignificant in the female model.

#### 3.3.2. Race/Ethnicity

The model fit was good (*χ* ^2^ = 1.47, *P* = .20; CFI = 1.00; RMSEA = .008). The Caucasian only model replicated the general model regarding significant predictor variables. Mixed messages and obesity-inherited were predictive of WP for Hispanics and Whites but not for Asians and Blacks. Obesity-behavior predicted WP for Blacks only. Information-seeking predicted WP for all groups except Asians. None of the variables predicted GH for Asians. Obesity-inherited and obesity-behavior predicted PH for Whites only. Information-seeking predicted PH for Hispanics and Whites but not Blacks. Obesity-behavior and PH predicted trying to lose weight for Whites only.

#### 3.3.3. Education

This model had good fit (*χ* ^2^ = .47, *P* = .76; CFI = 1.00; RMSEA = .000). Mixed messages significantly predicted WP for college graduates. Though nonsignificant in the general model, obesity-behavior positively predicted WP for participants with less than a high school education. Information-seeking predicted WP in all models except less than high school education. Mixed messages and obesity-inherited predicted PH for college graduates and the general model only. Obesity-behavior predicted PH for high school and college graduates only. Information-seeking did not significantly predict PH for any of the education-specific models. Regarding trying to lose weight, obesity-behavior was only significant for participants with some college education. Contrary to the general model, obesity-inherited was significantly positively predictive of trying to lose weight for high school graduates. Information-seeking and PH were predictive of trying to lose weight in all models except participants with less than a high school education.

#### 3.3.4. Weight Misperception

This model had good fit (*χ* ^2^ = 6.51, *P* < .001; CFI = .95; RMSEA = .028). Results of weight misperception models are presented in Table 4 and described in the following subsections.

*Overestimate*. For participants who overestimated their weight status, WP was positively predicted by mixed messages and information-seeking. PH was negatively predicted by mixed messages and WP and positively predicted by obesity-behavior. Trying to lose weight was positively predicted by information-seeking, WP, and PH.

*Correct Estimate*. For participants who correctly perceived their weight status, mixed messages, obesity-inherited, and information-seeking were positively related to WP. Mixed messages and WP were negatively related to PH, while information-seeking was positively related to PH. Trying to lose weight was positively predicted by WP, PH, information-seeking, and obesity-behavior.

*Underestimate*. Beliefs and information-seeking were unrelated to WP for participants who underestimated their weight status. Mixed messages were negatively related to PH, while information-seeking and WP were positively related to PH. WP and obesity-behavior were the only variables related to losing weight.

## 4. Discussion

### 4.1. Correlates of Weight Perception

WP and beliefs about the origins of obesity have far reaching potential public health implications since they may have a profound impact on individuals' obesogenic behaviors. The multinomial logistic regression provided promising results on the behaviors of those participants who tend to see themselves as overweight. Overweight participants being more likely to seek out information about diet, healthful eating, and PA on the internet suggest that they may recognize the need to do something to improve their weight status and are at least in the contemplation stage of change [25]. Overweight participants being more aware of recommended F and V may be reflective of increased information-seeking. It will be interesting to explore knowledge about other food groups and sizes of servings as it relates to perceived weight status. Further, the extent to which this knowledge translates into behavior should be explored in future studies.

As expected, the relationship between WP and PH was such that all groups perceived their GH as better than the overweight group. Though this is intuitive, it becomes problematic if people misperceive their weight, such that they may overestimate or underestimate their health and this may influence their decision to try to lose weight (see SEM models, Tables 3 and 4).

### 4.2. Tenets of HBM Predicting Decision to Lose Weight

The good fit observed across the tested models provides evidence of the role of beliefs about obesity, WP, and health perceptions in individuals' decisions and actions regarding weight management. Though the general model had great fit, we believed it was important to compare group models by gender, race/ethnicity, and education since there were significant within group variability across these variables. Additionally, because of the plethora of literature on weight misperceptions [8, 10, 11, 26], we believed that it was important to explore model differences based on the ability to correctly identify weight status.

Serdula and colleagues [27] researched weight loss attempts and strategies for controlling weight among adults. They found that women were more likely to try to lose weight than men across all sociodemographic categories, BMI predicted trying to lose weight, and individuals with higher education were more likely to try to lose weight. They also found that 90% of participants modified their diet and about two-thirds of participants engaged in physical activity to try to lose weight. This study builds on Serdula and colleagues' [27] findings via our exploration of the role of beliefs in this decision to lose weight. Beliefs that obesity was due to overeating and inactivity were only related to trying to lose weight for males, Whites, individuals with some college education, and participants who correctly identified their weight status in our study.

The predictive abilities of causal beliefs about obesity variables depict a trend of attribution biases among participants. Specifically, participants were more willing to identify their weight status as higher and perceived health as lower when they perceived obesity to be inherited and that there were mixed messages about the role of weight on health. However, when they believed obesity was due to inactivity and overeating, they perceived themselves to be healthier. Consequently, participants were more willing to admit to increased weight and poorer health when the causes could not be blamed on their behavior than when it could. This trend was most pronounced in females, Whites, and college graduates. Additionally, participants who correctly identified their weight status also had a similar trend with the exception of the relationship between obesity-inherited and general health.

Obesity-behavior was positively related to WP for Black participants and participants with less than a high school education; however these beliefs did not predict PH or losing weight for these participants. These findings suggest that there may be a disconnection between behavioral causes of obesity and the steps that Blacks and participants with less than a high school education take to try to lose weight. Obesity-behavior being insignificant in the models for Asians and Hispanics suggest that more may need to be done to make the connection between overeating and inactivity and health for these subpopulations.

Considering that, in the general literature, women are more likely to engage in weight management behaviors [28, 29], it was surprising that, in our sample, their beliefs about the role of overeating and inactivity in obesity had no significant influence on WP and trying to lose weight. The extent to which these differing beliefs and associations affect the gender differences in weight loss and maintenance success should be explored in future studies. Though we expected a negative relationship between obesity-inherited and trying to lose weight, the nonsignificance between these variables (except for high school graduates) is encouraging. As mentioned before, researchers are debating the usefulness of disclosing information about the genetic contributors to obesity to the general public for fear that this may hinder or discourage weight management efforts [15].

Regarding trying to lose weight, we expected mixed messages about obesity to have an overwhelming influence on this variable. However, mixed messages were unrelated to trying to lose weight in every model tested and were eventually set to 0 to preserve degrees of freedom. Surprisingly, mixed messages positively predicted WP and negatively predicted PH. Taken together, we can surmise that factors beyond beliefs, such as doctors' recommendations and mass media, about what causes obesity may play a role in individuals' taking behavioral measures to try to lose weight.

Cues to action in the form of seeking information on the internet in the last 12 months about diet, PA, and weight were positively related to WP, PH, and trying to lose weight. The positive relationship between WP and information-seeking complements our multinomial regression analyses which showed that this variable distinguishes overweight participants from all other classes of participants. It also supports research findings that suggest that individuals diagnosed with a disability or chronic disease are more likely to seek information on the internet about health issues including diet [30]. This is encouraging because it suggests that individuals, who may most likely benefit from knowledge on diet, PA, and weight, are seeking information and may be using it to try to lose weight. The findings that information-seeking was positively related to perceived health in several models of ours were surprising since we would expect participants with poorer health status to be more likely to seek health information [31, 32]. One possible explanation for this is that seeking information and possibly using the information may elevate people's actual health status as well as their perceptions of their health status. Noteworthy is the insignificant results found for participants with less than a high school education. In addition to having fewer resources for internet use, research has also shown that less educated individuals are more likely to use the internet for entertainment rather than information-seeking [33]. Therefore, increasing the health knowledge of this population may require more direct cues to action such as media campaigns, health literacy interventions, and targeted knowledge dissemination where the knowledge is disseminated in a relatable and entertaining manner.

The positive relationship between WP and trying to lose weight suggests that people who perceive their weight to be high were taking steps to manage their weight. The inverse relationship between WP and PH is also encouraging since it is an acknowledgement among participants that a higher weight status is not equivalent to good health status. It also provides evidence for the continued need to highlight the relationship between health and weight status.

The positive relationship between PH and trying to lose weight was surprising since we expected that participants who perceived their health status as lower would be more likely to try to lose weight. One possible explanation for these findings might be that the participants with lower health status may have health problems unrelated to weight therefore trying to lose weight may not help their condition. Another possible explanation is that similar to information-seeking, engaging in trying to lose weight improves perceived health status.

Particularly noteworthy among the weight misperception models are the differences in the variance explained. The variance explained for losing weight in the correct identification model was notably larger than that for the overestimation and underestimation of weight status models suggesting that the model used in this study may be missing key variables needed to better understand the decision to lose weight among those who do not correctly identify their weight status. Additionally, with the exception of the pathways for perceived health, the correct weight estimation model was identical to the general model (Table 3). This highlights the need to recognize WP as separate from BMI when studying and intervening on attitudes, beliefs, and behaviors for weight management.

### 4.3. Limitations and Future Directions

Our study is not without limitations. First, causal beliefs about obesity variables were restricted to single-items measures, hence compromising the reliability of our measurement. Future studies should improve the measurement of causal beliefs using multiple item scales with good internal consistency. This study was also restricted by the causal beliefs of obesity measured; other causes of obesity such as chronic health conditions and environmental contributions were not measured and may have significant impact on people's decision to lose weight. We assessed individuals' understanding of appropriate F and V and PA to promote health and healthy weight. However, other variables such as beliefs about caloric intake, portion control, and sedentary activity were not assessed. Future research should incorporate these variables to improve our understanding of individuals' perceptions regarding all aspects of diet/nutrition and PA in order to better inform interventions. This study was restricted by the wording used in the WP item. The item responses were not parallel to BMI categories; therefore the authors combined categories when comparing the misperception groups. The combining of categories may have led to some false positive and false negatives in each group. Additionally, BMI was calculated using participants self-reported height and weight and the extent to which participants accurately reported these variables would influence the validity of the weight misperception results. Last, this study was cross-sectional and although the HBM has predictive value, it is important that inferences about causality are not made. Future research using longitudinal data is necessary to determine the role of causal beliefs of obesity in decisions to lose weight.

## 5. Conclusions

Research has shown that self-perceptions, beliefs regarding weight status, and beliefs about the origins of obesity influence individuals' behaviors regarding their weight status. This study adds to the literature by exploring the relationship between WP and correlates of obesity beyond demographic variables. Additionally, this study uses tenets of the HBM to explore the potential role of beliefs about obesity in how people perceive their weight and health and how this in turn predicts decisions to lose weight. Research guided by theory has greater impact and more applicability to practice. Researchers and practitioners should attend to the demographic and weight misperception variability in attitudes and behavior regarding obesity and weight management in this study. These findings should be a basis for further inquiry in these subpopulations as well as inform targeted interventions. A noteworthy implication for practitioners is the insignificant role of internet information-seeking for participants with less than a high school education. Practitioners, as well as researchers, who rely heavily on internet campaigns and internet-based interventions, may need additional strategies to reach this vulnerable population.

## Figures and Tables

**Table 1:** Characteristics of study participants.

	*n*	%
Gender (missing = 5)		
Male	2894	38.8
Female	4557	61.2
Age (missing = 20)		
18–34	1075	14.5
35–39	490	6.6
40–44	578	7.8
45+	5293	71.2
Race/ethnicity (missing = 298)		
Hispanic	621	8.7
White	5434	75.9
Black	680	9.5
Asian	201	2.8
Non-Hispanic other	222	3.1
Education (missing = 159)		
Less than high school	683	9.4
High school graduate	1801	24.7
Some college	2185	29.9
College graduate	2628	38
Information-seeking (missing = 2506)		
Yes	1813	36.6
No	3137	63.4
Weight loss (missing = 28)		
Yes	4096	55.1
No	3332	44.9
Weight perception (missing = 28)		
Underweight	336	4.5
“Just right”	2177	29.3
Slightly overweight	2690	36.2
Overweight	2225	30
Weight misperception (missing = 237)		
Underestimated	849	11.8
Correctly estimated	5495	76.1
Overestimated	875	12.1
F and V recommendations (missing = 137)		
Less than 5	4155	56.8
5–9	2567	35.1
More than 9	597	8.2
PA recommendations (missing = 995)		
Sedentary (0 minutes)	58	.9
<Recommended (15–149)	2705	41.9
Recommended (150)	893	13.8
>Recommended (151–300)	1937	30
>300	868	13.4

**Table 2:** Multinomial regressions comparing participants in different weight classes' demographics, beliefs about obesity, and recommendations for fruits, vegetables, and physical activity.

	Overweight versus underweight		Overweight versus just right		Overweight versus slightly overweight	
	OR [95% CI]	*P*	OR [95% CI]	*P*	OR [95% CI]	*P*
Age	.97 [.95–.98]	<.001	.98 [.97–.98]	<.001	.99 [.98–.99]	<.001
Gender	.36 [.25–.52]	<.001	.55 [.45–.66]	<.001	.62 [.53–.73]	<.001
Race (White = ref)						
Hispanic	1.21 [.61–2.39]	.589	1.08 [.75–1.55]	.695	1.13 [.82–1.55]	.470
Black	1.36 [.75–2.49]	.313	.78 [.56–1.09]	.152	.81 [.61–.78]	.139
Asian	10.08 [4.48–22.68]	<.001	5.39 [2.88–10.09]	<.001	2.21 [1.17–4.16]	.014
Other	.50 [.12–2.15]	.351	.83 [.48–1.44]	.502	1.06 [.69–1.65]	.782
Education (college graduate = ref)						
<High school	1.77 [.72–4.32]	.212	.82 [.45–1.49]	.509	.99 [.60–1.61]	.953
High school graduate	.83 [.48–1.44]	.513	.81 [.63–1.04]	.096	.61 [.49–.76]	<.001
Some college	.94 [.61–1.43]	.767	.74 [.60–.90]	.003	.79 [.66–.94]	.007
Internet information-seeking	.41 [.27–.61]	<.001	.46 [.39–.56]	<.001	.66 [.57–.78]	<.001
Perceived health	1.95 [1.57–2.43]	<.001	2.93 [2.62–3.27]	<.001	1.86 [1.70–2.04]	<.001
Obesity-inherited	.77 [.62–.95]	.015	.80 [.72–.89]	<.001	.87 [.79–.96]	.005
Obesity-behavior	.88 [.64–1.21]	.428	1.00 [.85–1.18]	.959	.98 [.85–1.13]	.782
Mixed messages	.89 [.75–.1.06]	.183	.93 [.86–1.01]	.097	1.05 [.98–1.13]	.179
Recommended F and V (5–9 = ref)						
<Recommended	1.93 [1.28–2.92]	.002	1.50 [1.25–1.81]	<.001	1.58 [1.35–1.86]	<.001
>Recommended	2.95 [1.45–6.00]	.003	1.23 [.81–1.85]	.336	.86 [.58–1.27]	.448
Recommended PA (150 min = ref)						
None	5.41 [.91–32.06]	.063	.90 [.21–3.86]	.890	1.43 [.48–4.25]	.517
<Recommended	1.67 [.87–3.21]	.122	1.16 [.90–1.48]	.257	1.23 [.99–1.53]	.061
>Recommended	1.83 [.94–3.59]	.078	1.22 [.94–1.60]	.134	1.21 [.96–1.53]	.102
>300	2.41 [1.11–5.27]	.027	1.53 [1.07–2.18]	.020	1.56 [1.14–2.14]	.005

Note. OR = odds ratio; CI = confidence interval; ref = reference group; F and V = fruits and vegetables; PA = physical activity.

**Table 3:** Results of general and group structural equation models predicting participants' decisions to lose weight.

	General *χ* ^2^ = .30, *P* = .59; CFI = 1.00; RMSEA = .000	Gender *χ* ^2^ = 2.81, *P* = .06; CFI = .99; RMSEA = .016		Race/ethnicity *χ* ^2^ = 1.47, *P* = .20; CFI = 1.00; RMSEA = .008				Education *χ* ^2^ = .47, *P* = .76; CFI = 1.00; RMSEA = .000			
		Male	Female	Hispanic	Black	Asian	White	<HS	HS	Some college	College
WP											
MM	.04∗∗∗	.04∗	.04∗	.12∗∗	.03	.06	.03∗	.05	.01	.02	.06∗∗
Behavior	.02^†^	.04∗	.01	−.02	.12∗∗	.13^†^	.01	.08∗	.03	.01	−.00
Inherited	.07∗∗∗	.04∗	.09∗∗∗	.13∗∗	−.00	−.09	.08∗∗∗	.09∗	.10∗∗∗	.06∗∗	.07∗∗∗
Info-seek	.12∗∗∗	.09∗∗∗	.13∗∗∗	.13∗	.20∗∗∗	.10	.12∗∗∗	−.03	.14∗∗∗	.12∗∗∗	.12∗∗∗

Perceived health											
MM	−.10∗∗∗	−.08∗∗∗	−.11∗∗∗	−.16∗∗∗	−.09∗	−.06	−.08∗∗∗	−.00	−.02	−.04^†^	−.08∗∗∗
Behavior	.04∗∗∗	.03	.05∗∗∗	.07^†^	−.01	−.07	.04∗∗	−.03	.08∗∗	.00	.04∗
Inherited	−.03∗	.00	−.05∗∗∗	.02	−.02	−.06	−.03∗	−.05	−.00	−.02	−.07∗∗∗
Info-seek	.05∗∗∗	.08∗∗∗	.04∗	.15∗∗	.06	−.14^†^	.06∗∗∗	−.03^†^	−.01	.04	.04^†^
WP	−.22∗∗∗	−.21∗∗∗	−.22∗∗∗	−.25∗∗∗	−.23∗∗∗	−.03	−.23∗∗∗	−.16∗∗∗	−.19∗∗∗	−.21∗∗∗	−.29∗∗∗

Lose weight											
Behavior	.03∗∗	.07∗∗∗	.01	−.03	.04	.05	.04∗∗	.02	.03	.04∗	.02
Inherited	.01	−.01	.02^†^	−.01	.02	.08	.01	−.03	.04∗	.02	.00
Info-seek	.17∗∗∗	.13∗∗∗	.19∗∗∗	.10∗	.13∗∗	.13∗	.18∗∗∗	.06	.13∗∗∗	.19∗∗∗	.17∗∗∗
WP	.45∗∗∗	.44∗∗∗	.44∗∗∗	.45∗∗∗	.54∗∗∗	.44∗∗∗	.44∗∗∗	.47∗∗∗	.46∗∗∗	.45∗∗∗	.44∗∗∗
Perceived health	.07∗∗∗	.05∗∗	.09∗∗∗	.04	.06^†^	.09	.08∗∗∗	−.01	.05∗	.06∗∗∗	.07∗∗∗

*R* ^2^											
WP	.02	.01	.03	.05	.06	.04	.02	.02	.03	.02	.02
Perceived health	.06	.06	.07	.11	.06	.04	.07	.05	.04	.05	.11
Lose Weight	.25	.23	.25	.21	.33	.24	.24	.23	.25	.25	.23

Correlations											
MM × Beh	−.03∗	−.02	−.03∗	.04	−.03	−.08	−.03∗	.05	.03	−.03	−.05∗∗
MM × Inh	.10∗∗∗	.11∗∗∗	.09∗∗∗	.01	.13∗∗∗	.09	.11∗∗∗	.07^†^	.07∗∗	.07∗∗∗	.15∗∗∗
MM × Info	−.07∗∗∗	−.06∗∗	−.07∗∗∗	.07	−.14∗∗	.11	−.07∗∗∗	.07	−.04	−.03	−.05∗
Beh × Info	.05∗∗	.01	.07∗∗∗	.12∗	.09^†^	.13	.03^†^	.10	.06^†^	.04	.02
Inh × Info	.01	.00	.02	−.03	.10^†^	.03	.01	.08	−.04	.08∗∗	−.02
Inh × Beh	−.02	−.01	−.02	.05	.08^†^	.19∗∗∗	−.06∗∗∗	.07^†^	.01	−.04^†^	−.06∗∗

Behavior: obesity due to behavior; inherited: obesity is inherited; MM: mixed messages about weight and health; Beh: behavior; HS: high school; info-seek: information-seeking; info: information-seeking; Inh: inherited; WP: weight perception; CFI: Confirmatory Fit Index; RMSEA: Root Mean Square Error of Approximation.

^†^≤.10;∗≤.05; ∗∗≤.01; ∗∗∗≤.001.

**Table 4:** Results of structural equation models predicting participants' decisions to lose weight based on weight misperception grouping.

	*χ* ^2^ = 6.51, *P* < .000; CFI = .95; RMSEA = .028		
	Overestimate	Correct	Underestimate
WP			
Gender	.03	.03∗	−.11∗∗
Race/ethnicity	−.04	−.05∗∗	−.02
MM	.09∗	.06∗∗∗	.07^†^
Behavior	−.01	.01	.01
Inherited	.01	.07∗∗∗	−.00
Info-seek	.08∗	.12∗∗∗	.04

Perceived health			
Gender	.01	−.01	−.07
Race/ethnicity	.10∗∗	.10∗∗∗	.05∗∗∗
MM	−.16∗∗∗	−.07∗∗∗	−.07∗
Behavior	.09∗∗	.02	.04
Inherited	−.03	−.02	−.06^†^
Info-seek	.07^†^	.05∗∗	.12∗
WP	−.10∗∗	−.29∗∗∗	.13∗∗∗

Lose weight			
Gender	.12∗∗∗	.10∗∗∗	.07∗
Race/ethnicity	.01	.00	.05
Behavior	.03	.03∗	.07∗
Inherited	.02	.00	−.00
Info-seek	.23∗∗∗	.17∗∗∗	.07
WP	.21∗∗∗	.44∗∗∗	.23∗∗∗
Perceived health	.10∗∗	.07∗∗∗	.05

*R* ^2^			
WP	.02	.03	.02
Perceived health	.06	.11	.06
Lose weight	.13	.24	.07

Behavior: obesity due to behavior; inherited: obesity is inherited; MM: mixed messages about weight and health; info-seek: information-seeking; WP: weight perception; CFI: Confirmatory Fit Index; RMSEA: Root Mean Square Error of Approximation.

^†^≤.10; ∗≤.05; ∗∗≤.01; ∗∗∗≤.001.

## References

[1] Caballero B. (2007). The global epidemic of obesity: an overview. *Epidemiologic Reviews*.

[2] Flegal K. M., Carroll M. D., Ogden C. L., Johnson C. L. (2002). Prevalence and trends in obesity among US adults, 1999-2000. *The Journal of the American Medical Association*.

[3] Ogden C. L., Carroll M. D., Curtin L. R., McDowell M. A., Tabak C. J., Flegal K. M. (2006). Prevalence of overweight and obesity in the United States, 1999–2004. *The Journal of the American Medical Association*.

[4] Flegal K. M., Carroll M. D., Ogden C. L., Curtin L. R. (2010). Prevalence and trends in obesity among US adults, 1999–2008. *Journal of the American Medical Association*.

[5] Knudsen N., Laurberg P., Rasmussen L. B. (2005). Small differences in thyroid function may be important for body mass index and the occurrence of obesity in the population. *The Journal of Clinical Endocrinology and Metabolism*.

[6] Graff M., North K. E., Monda K. L. (2011). The combined influence of genetic factors and sedentary activity on body mass changes from adolescence to young adulthood: the National Longitudinal Adolescent Health Study. *Diabetes/Metabolism Research and Reviews*.

[7] Chang V. W., Christakis N. A. (2003). Self-perception of weight appropriateness in the United States. *American Journal of Preventive Medicine*.

[8] Duncan D. T., Wolin K. Y., Scharoun-Lee M., Ding E. L., Warner E. T., Bennett G. G. (2011). Does perception equal reality? Weight misperception in relation to weight-related attitudes and behaviors among overweight and obese US adults. *International Journal of Behavioral Nutrition and Physical Activity*.

[9] McGrath Davis A., James R. L., Curtis M. R., Felts S. M., Daley C. M. (2008). Pediatric obesity attitudes, services, and information among rural parents: a qualitative study. *Obesity*.

[10] Dorsey R. R., Eberhardt M. S., Ogden C. L. (2009). Racial/ethnic differences in weight perception. *Obesity*.

[11] Bennett G. G., Wolin K. Y. (2006). Satisfied or unaware? Racial differences in perceived weight status. *International Journal of Behavioral Nutrition and Physical Activity*.

[12] Kuchler F., Variyam J. N. (2003). Mistakes were made: misperception as a barrier to reducing overweight. *International Journal of Obesity*.

[13] Rahman M., Berenson A. B. (2010). Self-perception of weight and its association with weight-related behaviors in young, reproductive-aged women. *Obstetrics and Gynecology*.

[14] Anderson L. A., Eyler A. A., Galuska D. A., Brown D. R., Brownson R. C. (2002). Relationship of satisfaction with body size and trying to lose weight in a national survey of overweight and obese women aged 40 and older, United States. *Preventive Medicine*.

[15] Wang C., Coups E. J. (2010). Causal beliefs about obesity and associated health behaviors: results from a population-based survey. *International Journal of Behavioral Nutrition and Physical Activity*.

[16] Razquin C., Marti A., Martinez J. A. (2011). Evidences on three relevant obesogenes: MC4R, FTO and PPAR*γ*. Approaches for personalized nutrition. *Molecular Nutrition and Food Research*.

[17] Rankinen T., Zuberi A., Chagnon Y. C. (2006). The human obesity gene map: the 2005 update. *Obesity*.

[18] Becker M. H., Maiman L. A., Kirscht J. P., Haefner D. P., Drachman R. H. (1977). The health belief model and prediction of dietary compliance: a field experiment. *Journal of Health and Social Behavior*.

[19] Lewin K., Dembo T., Festinger L., Sears P. S., Hunt J. M. (1944). Level of aspiration. *Personality and the Behavior Disorders: A Handbook Based on Experimental and Clinical Research*.

[20] Rosenstock I. M. (1966). Why people use health services. *The Milbank Memorial Fund quarterly*.

[21] Rosenstock I. M. (1974). The health belief model and preventive health behavior. *Health Education & Behavior*.

[22] Cantor D., Coa K., Crystal-Mansour S., Davis T., Dipko S., Sigman R. (2009). *Health Information National Trends Survey 2007: Final Report*.

[23] http://www.exerciseismedicine.org/documents/PublicActionGuide_LR.pdf.

[24] (2007). *SPSS for Windows*.

[25] Prochaska J. O., DiClemente C. C., Norcross J. C. (1992). In search of how people change: applications to addictive behaviors. *The American Psychologist*.

[26] Burke M. A., Heiland F. W., Nadler C. M. (2010). From “Overweight” to “About Right”: evidence of a generational shift in body weight norms. *Obesity*.

[27] Serdula M. K., Mokdad A. H., Williamson D. F., Galuska D. A., Mendlein J. M., Heath G. W. (1999). Prevalence of attempting weight loss and strategies for controlling weight. *Journal of the American Medical Association*.

[28] Fayet F., Petocz P., Samman S. (2012). Prevalence and correlates of dieting in college women: a cross sectional study. *International Journal of Women's Health*.

[29] Kruger J., Galuska D. A., Serdula M. K., Jones D. A. (2004). Attempting to lose weight: Specific practices among U.S. Adults. *The American Journal of Preventive Medicine*.

[30] Goldner M. (2006). How health status impacts the types of information consumers seek online. *Information Communication and Society*.

[31] Reinfeld-Kirkman N., Kalucy E., Roeger L. (2010). The relationship between self-reported health status and the increasing likelihood of South Australians seeking Internet health information. *Australian and New Zealand Journal of Public Health*.

[32] Renahy E., Parizot I., Chauvin P. (2010). Determinants of the frequency of online health information seeking: results of a web-based survey conducted in France in 2007. *Informatics for Health and Social Care*.

[33] Bonfadelli H. (2002). The Internet and knowledge gaps: a theoretical and empirical investigation. *European Journal of Communication*.

